# Improved Statistical Tools Reveal Many Linked Loci

**DOI:** 10.1371/journal.pbio.0030294

**Published:** 2005-07-26

Using traditional statistical tools to analyze the modern wealth of biological data is a bit like trying to move a muscle car with a buggy whip—you're not likely to get anywhere very fast. The problem is perhaps most acute in the quest to understand how genes interact to regulate one another's expression. The amount of RNA made by any one gene is likely influenced by DNA at dozens of loci, or locations around the genome. Such loci are often situated within genes that participate in the same pathway as the gene being influenced, and a central goal is to understand this network of mutually influential genes and loci. Consider piecing together this puzzle for each of the many thousands of genes and many thousands of potentially influential loci, and the old analytical tools simply can't keep up. In this issue of *PLoS Biology*, John Storey and colleagues tackle the challenge with a new approach.

The authors began by mating two strains of yeast that have minor differences in their DNA at more than 3,000 loci—creating over 3,000 markers—and then tracking the inheritance of these markers in the yeast offspring. Because the two genomes randomly reshuffle upon mating, any single offspring will contain some random combination of marker outcomes from each parent. The authors also examined the amount of RNA produced by over 6,000 individual genes in each offspring. The next step was to determine how these two large sets of data—variations at specific loci and variations in expression of specific genes—were correlated.

Straightforward statistical tests performed on each gene's expression revealed the single most influential location in the DNA. But such tests don't reveal the more complicated reality that any single gene is likely to be influenced by more than one locus. Linking expression of a single gene (or “expression trait,” in genetic parlance) to more than one locus has been stymied by the inability of conventional statistical approaches to cope with the mountains of data involved. Not only is an exhaustive pair-by-pair testing of all possible interactions computationally demanding, but it can also be very difficult to distinguish whether elevated expression is due to one or both of the loci being tested. The problem becomes exponentially harder as more potentially linked loci are tested. [Fig pbio-0030294-g001]

**Figure pbio-0030294-g001:**
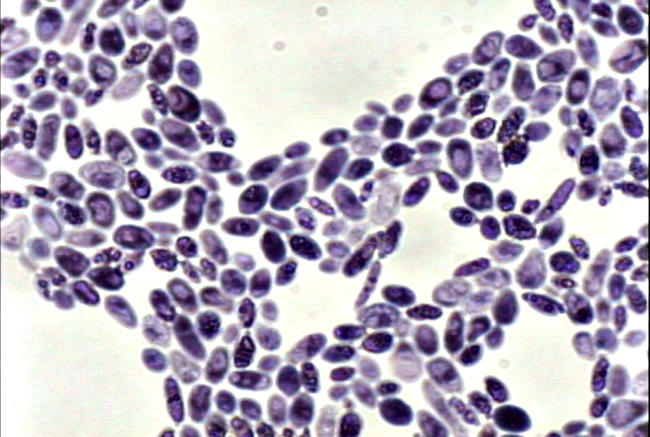
To unravel the tangled web of interactions between yeast genes, researchers bred two distinct yeast strains and statistically analyzed how gene expression varied between their offspring (Photo: David Byres)

To overcome the limitations of standard approaches, Storey et al. used a novel statistical approach that exploited what they had the most of—data. They began by determining the single most significant locus for each expression trait. They then moved on to the next most significant locus for that trait, but tested its linkage (that is, its influence on expression) with the assumption that the first locus was also linked. The ability to assign a “probability of linkage” to the first locus greatly simplified the calculations for the subsequent locus, reducing by almost a thousand-fold the number of possibilities that needed to be tested.

As opposed to standard methods, the authors show that their approach is able to assess true *joint* linkage of two loci to an expression trait, while requiring substantially less computation. In addition, they found that about one in seven expression traits is controlled by “epistatic,” or hierarchical, relationships among the two loci, while the standard method revealed none. This method can be adapted to search for even larger numbers of linked loci, to provide insights into the many interlocking pathways that make up the gene regulatory network, and ultimately result in the organism itself.

